# Positive Regulation of S-Adenosylmethionine on Chondrocytic Differentiation via Stimulation of Polyamine Production and the Gene Expression of Chondrogenic Differentiation Factors

**DOI:** 10.3390/ijms242417294

**Published:** 2023-12-09

**Authors:** Loc Dinh Hoang, Eriko Aoyama, Miki Hiasa, Hiroshi Omote, Satoshi Kubota, Takuo Kuboki, Masaharu Takigawa

**Affiliations:** 1 Advanced Research Center for Oral and Craniofacial Sciences (ARCOCS), Okayama University Graduate School of Medicine, Dentistry and Pharmaceutical Sciences, Okayama 700-8525, Japan; pkph12us@s.okayama-u.ac.jp; 2 Department of Oral Rehabilitation and Regenerative Medicine, Okayama University Graduate School of Medicine, Dentistry and Pharmaceutical Sciences, Okayama 700-8525, Japan; kuboki@md.okayama-u.ac.jp; 3 Laboratory of Membrane Biochemistry, Okayama University Graduate School of Medicine, Dentistry and Pharmaceutical Sciences, Okayama 700-0082, Japan; hiasa@okayama-u.ac.jp (M.H.); omote-h@okayama-u.ac.jp (H.O.); 4 Department of Biochemistry and Molecular Dentistry, Okayama University Graduate School of Medicine, Dentistry and Pharmaceutical Sciences, Okayama 700-8525, Japan; kubota1@md.okayama-u.ac.jp

**Keywords:** S-adenosylmethionine, chondrocyte differentiation, CCN2, polyamine, ODC, gene expression

## Abstract

S-adenosylmethionine (SAM) is considered to be a useful therapeutic agent for degenerative cartilage diseases, although its mechanism is not clear. We previously found that polyamines stimulate the expression of differentiated phenotype of chondrocytes. We also found that the cellular communication network factor 2 (CCN2) played a huge role in the proliferation and differentiation of chondrocytes. Therefore, we hypothesized that polyamines and CCN2 could be involved in the chondroprotective action of SAM. In this study, we initially found that exogenous SAM enhanced proteoglycan production but not cell proliferation in human chondrocyte-like cell line-2/8 (HCS-2/8) cells. Moreover, SAM enhanced gene expression of cartilage-specific matrix (aggrecan and type II collagen), Sry-Box transcription factor 9 (SOX9), CCN2, and chondroitin sulfate biosynthetic enzymes. The blockade of the methionine adenosyltransferase 2A (MAT2A) enzyme catalyzing intracellular SAM biosynthesis restrained the effect of SAM on chondrocytes. The polyamine level in chondrocytes was higher in SAM-treated culture than control culture. Additionally, Alcian blue staining and RT-qPCR indicated that the effects of SAM on the production and gene expression of aggrecan were reduced by the inhibition of polyamine synthesis. These results suggest that the stimulation of polyamine synthesis and gene expression of chondrogenic differentiation factors, such as CCN2, account for the mechanism underlying the action of SAM on chondrocytes.

## 1. Introduction

S-adenosylmethionine (SAM) is a crucial intermediate metabolite resulting from the catalytic processes of methionine and ATP by methionine adenosyltransferase [1]. Although SAM was initially synthesized in 1940 [2], it gained significant attention only after the chemical structure was described in 1953 [3]. Over the past 50 years, extensive research has been conducted which suggests SAM as a potential tool for the management of various disorders. SAM is currently marketed in the United States as a dietary supplement and a viable alternative for treating depression [4,5,6], osteoarthritis [7], and liver diseases [8,9].

Clinical trials have indicated that SAM may be an effective option for addressing osteoarthritis [10,11,12,13]. Specifically, it showed promising results in stimulating proteoglycan synthesis and secretion in cultured human chondrocytes [14,15]. SAM is recognized as the primary methyl donor in various biological processes, including DNA methylation, polyamine synthesis, and transsulfuration [16]. Despite the impressive safety and potential benefits of SAM [7], the exact mechanism underlying its effectiveness in the management of osteoarthritis remains incompletely understood.

Polyamines are small organic compounds containing multiple amines and are known to be essential for cell growth, differentiation, and apoptosis [17,18,19]. In mammalian cells, three types of polyamines, putrescine, spermine, and spermidine, are synthesized. The first polyamine putrescine is converted from L-ornithine by ornithine decarboxylase (ODC), a rate-limiting enzyme of polyamine synthesis. Spermidine and spermine are then synthesized by sequential addition of aminopropyl groups of decarboxylated SAM (dcSAM) to each end of the putrescine [20,21]. Interestingly, spermidine and spermine produced with SAM by transaminopropylation have been reported to enhance chondrogenesis, increase type II collagen deposition, and stimulate chondrocyte terminal differentiation [22]. We previously reported that the induction of ODC, followed by an increase in polyamine levels in primary cultured rabbit growth cartilage cells stimulated by differentiation factors, such as parathyroid hormone (PTH) and cAMP derivatives precedes the increased expression of the differentiated phenotype of chondrocytes [23,24], and that the exposure of the growth chondrocytes to exogenous polyamine increases glycosaminoglycan (GAG) production, a marker of chondrocyte differentiation [25]. However, whether SAM is clinically effective against osteoarthritis through regulation of polyamine synthesis has not been investigated.

In addition to the involvement of polyamine in SAM’s mechanism of action, this study also focused on the gene expression of chondrocyte markers and chondrocyte differentiation factors, including cellular communication network factor 2 (CCN2). CCN2, a classical member of the CCN family of matricellular proteins, also known as CTGF, recently renamed as cellular communication network factor 2, was found to have a huge influence on chondrocytes. Having a special structure constructed by four distinct modules (insulin-like growth factor binding protein-like (IGFBP), von Willebrand factor type C repeat (VWC), thrombospondin 1 type 1 repeat (TSP1), and C-terminal cystine knot (CT) modules) [26,27,28,29], CCN2 possesses a vast binding capacity to an array of molecules. These binding counterparts include an aggrecan core protein, heparan sulfate proteoglycans, cell-surface receptors, and growth factors [26,27,29]. These multiple interactions confer the massive influence of CCN2 in a variety of chondrocyte-related signaling pathways [26,28,30]. More importantly, CCN2 has been shown to stimulate the proliferation and differentiation of chondrocytes towards the terminal state in the growth plate, but did not promote chondrocyte hypertrophic differentiation in articular chondrocytes [29]. Such evidence unequivocally demonstrates that CCN2 must play the role of orchestra conductor in the mechanisms that directly drive chondrocytes toward their distinct destinations with different functionalities in the in articular cartilage and growth plates.

The mechanism of SAM’s effects on chondrocytes are not fully understood. We hypothesized that the metabolic pathways regulated by SAM may induce CCN2 and regulate polyamine synthesis in chondrocytes, thereby promoting chondrocyte differentiation. We herein report that SAM positively regulates chondrocyte differentiation and plays an important role in cartilage protection via the polyamine synthesis pathway and the gene expression of several chondrogenic markers and growth factors.

## 2. Results

### 2.1. SAM Enhances Aggrecan Production, but Negatively Regulates Cell Proliferation of Chondrocytic HCS-2/8 Cells

**Figure 1:**
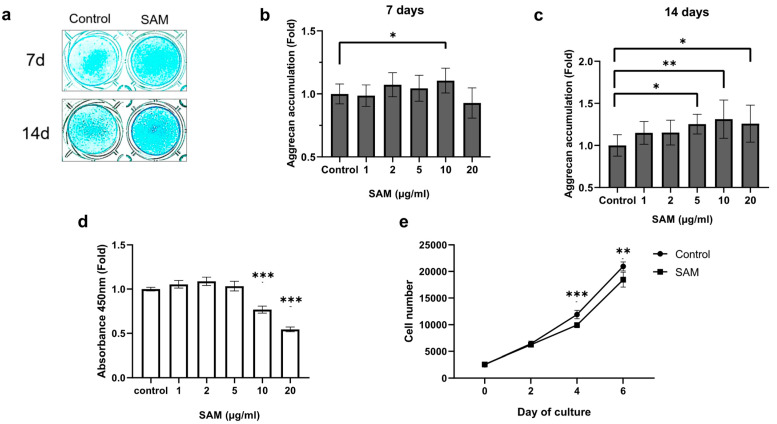
SAM enhances aggrecan production in chondrocytes, but negatively regulated cell proliferation in chondrocytes. Human chondrocyte-like cell line-2/8 (HCS-2/8) cells (1 × 10^5^ cells/well) were inoculated in 24-well plates and allowed to adhere for 24 h. Subsequently, S-adenosylmethionine (SAM) was added to culture at the indicated concentrations and kept incubated for 7 or 14 days. (**a**) Alcian blue staining revealed an increase of aggrecan accumulation in SAM-treated groups. (**b**,**c**) Quantitative measurement of Alcian blue staining on HCS-2/8 cells cultured with 7-day (**b**) and 14-day (**c**) of SAM stimulation showed that the addition of SAM significantly enhanced aggrecan production in HCS-2/8 cells (values are represent the fold-changes relative to a non-treated group, one-way ANOVA, Dunnett, n = 12). (**d**) Cell counting assay in cultures with several concentrations of SAM. HCS-2/8 cells (3000 cells/well) were inoculated in 96-well plates and allowed to adhere for 24 h. Cells were treated with the indicated concentrations of SAM for 2 days, and cell viability was measured with a WST-8 assay kit, as described in the Materials and Methods. The Y axis shows the relative ratio of absorbance to control that was obtained at a wavelength of 450 nm (ratio = 1) (one-way ANOVA, Dunnett, *** *p* < 0.005, n = 3). (**e**) Counting the number of cells cultured with SAM at several time points. Cells were treated with 10 μg/mL SAM for the indicated days and stained with DAPI. The number of nuclei was counted as the number of the cells using a Cellomics Array Scan^TM^ High Content Screening System. (The numbers of cells in the SAM-treated group and control group at each time point were compared using Welch’s t test, * *p* < 0.05, ** *p* < 0.01, *** *p* < 0.005, n = 10).

To confirm that SAM promotes proteoglycan synthesis in chondrocytes, Alcian blue staining of the HCS-2/8 cell layers was conducted. As shown in Figure 1, cell culture with exogenous SAM for 7 or 14 days enhanced Alcian blue staining (Figure 1a). The absorbance of the extract from cells indicated that treatment with SAM (10 μg/mL) resulted in an average increase of 10.6% in aggrecan accumulation on the 7th day (Figure 1b), and an average increase of 31.3% on the 14th day (Figure 1c). Similar experiments were conducted on rat chondrosarcoma derived cell line (RCS) cells, and we also found that 50 and 100 μg/mL SAM induced aggrecan accumulation, with an average increase of 15% and 28.3% after 7 days of culture, respectively (Supplementary Figure S1a,b).

Next, to determine whether the aggrecan accumulation caused by SAM is due to cell proliferation, we utilized the WST-8 assay to measure the viability of HCS-2/8 cells in the presence of SAM. The results in Figure 1d show that SAM did not promote the proliferation of HCS-2/8 cells. Additionally, the highest concentration of SAM (10 μg/mL), which was effective in stimulating aggrecan accumulation (Figure 1a–c), had a negative effect on cell proliferation (Figure 1d). To confirm this finding, we utilized a high-content image screening system to count the number of cells in each well in the presence of SAM at 10 μg/mL within the indicated time. The number of cells in the SAM-treated group was lower than that in the control group (Figure 1e). This result was consistent with that obtained from the WST-8 assay (Figure 1d). Similar experiments were conducted on RCS cells, and similar trends were observed. (Supplementary Figure S1c,d). These findings indicate that exogenous SAM enhances proteoglycan production in chondrocyte cell lines, regardless of cell proliferation.

### 2.2. SAM Enhances the Gene Expression of Cartilage-Specific Markers, Chondrogenesis-Associated Factors, and Chondroitin Sulfate Synthesis-Involved Enzymes in HCS-2/8 Cells

**Figure 2:**
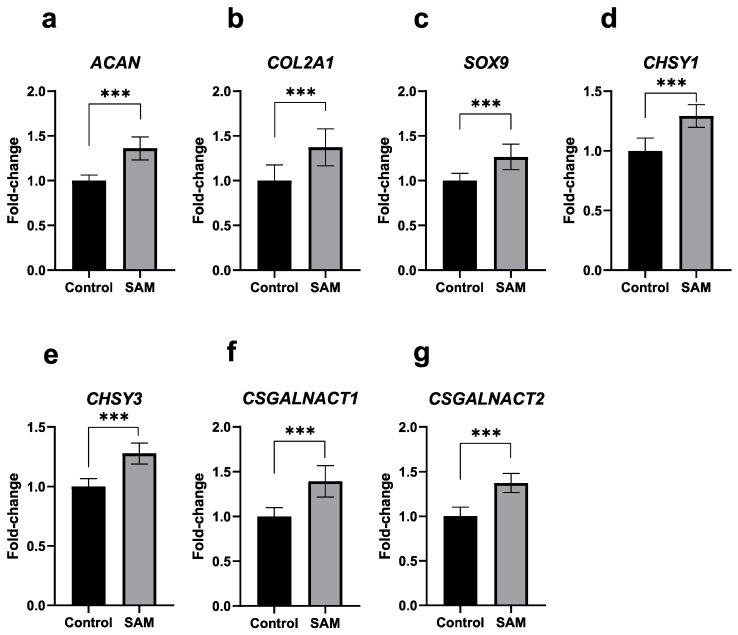
SAM enhances the gene expression of cartilage-specific markers, chondrogenesis associated factors, and enzymes involved in chondroitin sulfate synthesis in chondrocytes. HCS-2/8 cells were placed in 6-well plates at a density of 6 × 10^5^ cells/well and incubated with SAM at a concentration of 10 μg/mL. Three days later, total RNAs were extracted from the cells and subjected to RT-qPCR to detect the effects of SAM stimulation on the gene expression. The results demonstrated that the addition of SAM enhanced the gene expression of cartilage-specific markers: *ACAN* (**a**), *COL2A1* (**b**); a chondrogenesis-associated factor: *SOX9* and enzymes involved in chondroitin sulfate synthesis: (**c**); *CHSY1* (**d**); *CHSY3* (**e**); *CSGALNACT1* (**f**) *CSGALNACT2* (**g**) (values represent the fold-change relative to a non-treated group as control, Welch’s *t*-test, *** *p* < 0.005, n = 9).

To understand the effects of SAM on chondrocytic gene expression, we used RT-qPCR to analyze HCS-2/8 cells incubated with SAM for 3 days. The results in Figure 2 show that mRNA level of both *ACAN* and *COL2A1* increased due to the addition of SAM (Figure 2a,b). Furthermore, we also found that the gene expression of Sry-Box transcription factor 9 (SOX9), an important factor that plays a major role in the early stages of chondrogenesis [29,31], was enhanced by SAM (Figure 2c). In addition, we investigated whether SAM could stimulate the gene expression of glycosyltransferase enzymes involved in the production of chondroitin sulfate chains in cartilage; namely, chondroitin sulfate synthase 1 (CHSY1), chondroitin sulfate synthase 3 (CHSY3), chondroitin sulfate N-acetylgalactosaminyltransferase 1 (CSGALNACT1), and chondroitin sulfate N-acetylgalactosaminyltransferase 2 (CSGALNACT2), or not. Interestingly, exogenous SAM significantly enhanced the expression of HCS-2/8 cells (Figure 2d–g). A similar tendency was observed in the RCS cells (Supplementary Figure S2a–f).

### 2.3. Inhibition of Intracellular SAM Synthesis Impedes Aggrecan Accumulation in HCS-2/8 Cells

**Figure 3:**
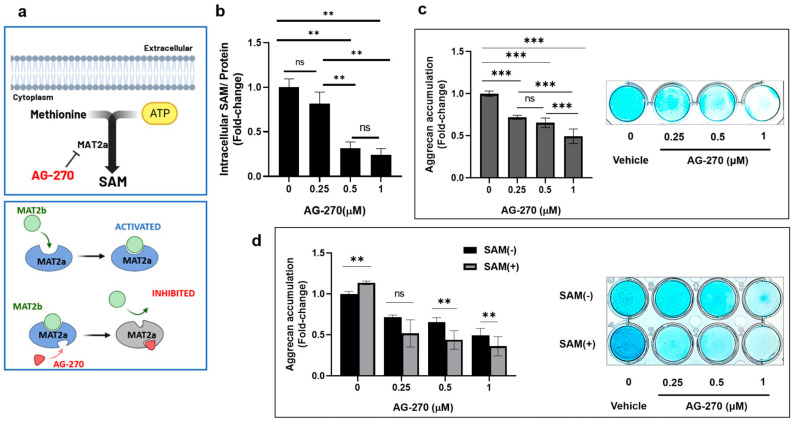
The inhibition of intracellular SAM synthesis impedes aggrecan accumulation in HCS-2/8 chondrocytes**.** (**a**) A diagram of AG-270 inhibiting methionine adenosyltransferase 2A (MAT2A), an enzyme catalyzing the production of SAM from methionine and ATP. (**b**) AG-270 pretreatment decreased intracellular SAM levels in HCS-2/8 cells. Cells were treated with AG-270 at the indicated concentrations for 3 days and the cell lysates were subjected to an ELISA (one-way ANOVA, Dunnett, ** *p* < 0.01, n = 3). (**c**) AG-270 pretreatment suppressed aggrecan production in HCS-2/8 cells. Cells were treated with AG-270 at the indicated concentrations for 14 days and then cell layers were stained by Alcian blue (one-way ANOVA, Dunnett, *** *p* < 0.005, n = 6). The images show the representative result. (**d**) The addition of SAM aggravated negative effect of AG-270 on aggrecan production. HCS-2/8 cells were cultured with AG-270/vehicle at the indicated concentration with/without SAM (10 μg/mL) for 14 days and then cell layers were stained with Alcian blue (two-way ANOVA, Tukey, ** *p* < 0.01, n = 6). ns = not significant. The images show the representative result. In all experiments, values represent the fold-change relative to non-treated groups (ratio = 1).

To confirm the effect of intracellular SAM on proteoglycan production in chondrocytes, we investigated whether the inhibition of internal SAM biosynthesis affects the aggrecan accumulation and gene expression in chondrocytes and whether exogenous SAM reverses the inhibitory effect, or not. In this study, we used AG-270, which inhibits the activity of MAT2A, the primary enzyme responsible for intracellular SAM biosynthesis (Figure 3a). As anticipated, the use of AG-270 in HCS-2/8 cells not only led to a significant decrease in intracellular SAM levels (Figure 3b), but also resulted in a reduction in aggrecan accumulation (Figure 3c). Pre-treatment with AG-270 (1 μM) drastically reduced the aggrecan accumulation by 53% in HCS-2/8 cells in comparison to the non-treated groups, and external SAM was not able to restore it (Figure 3d).

### 2.4. Inhibition of Intracellular SAM Synthesis Decreases the Gene Expression of Cartilage-Related Factors

**Figure 4:**
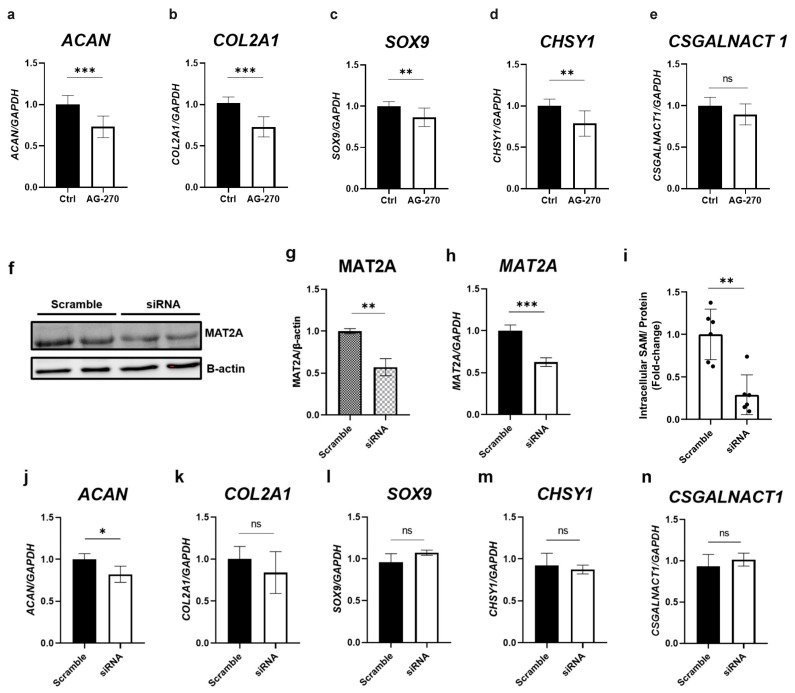
The inhibition of intracellular SAM synthesis decreases the gene expression of cartilage-related factors. HCS-2/8 cells were seeded in 6-well plates at 6 × 10^5^ cells/well and allowed to adhere for 24 h prior to incubation with inhibitor or siRNA, as described in “Materials and Methods.” (**a**–**e**) The effect of AG-270 on the gene expression of the following cartilage-related factors were analyzed by RT-qPCR: (**a**) *ACAN*, (**b**) *COL2A1*, (**c**) *SOX9*, (**d**) *CHSY1*, (**e**) *CSGALNACT1* (n = 9). (**f**,**g**) Western blotting and (**h**) RT-qPCR confirmed the efficiency of knockdown of siRNA targeting MAT2A (n = 5). (**i**) The amount of intracellular SAM was significantly decreased by the knockdown of MAT2A (n = 6). (**j**–**n**) The gene expression of cartilage-related factors under the effect of MAT2A knockdown: (**j**) *ACAN*, (**k**) *COL2A1*, (**l**) *SOX9*, (**m**) *CHSY1*, and (**n**) *CSGALNACT1* (n = 5). All data are presented as the mean ± SD of fold-change relative to the control group (Welch’s *t*-test, * *p* < 0.05, ** *p* < 0.01, *** *p* < 0.005). ns = not significant.

To further investigate the role of SAM in the gene expression of cartilage-related factors, RT-qPCR was performed using RNA samples from cells treated with AG-270. As shown in Figure 4a,b,d, the inhibition of MAT2A resulted in a significant decrease in the gene expression of *ACAN*, *COL2A1*, and *CHSY1*, while the gene expression of *SOX9* and *CSGALNACT1* tended to decrease (Figure 4c,e).

To confirm these results, we analyzed the gene expression in MAT2A knockdown cells using a siRNA system. The suppression of protein levels (Figure 4f,g) and the gene expression of MAT2A (Figure 4h) confirmed the knockdown efficiency of siRNA transfection. Additionally, SAM ELISA detected that the amount of intracellular SAM was decreased by the knockdown of MAT2A. Consequently, we found that MAT2A knockdown led to a significant decrease in the gene expression of *ACAN*, and likely *COL2A1* (Figure 4j,k). However, we did not observe any changes in the expression of *SOX9, CHSY1,* or *CSGALNACT1* (Figure 4l–n). 

### 2.5. SAM Has Positive Effects on the Gene Expression and Protein Level of CCN2 in HCS-2/8 Cells

**Figure 5:**
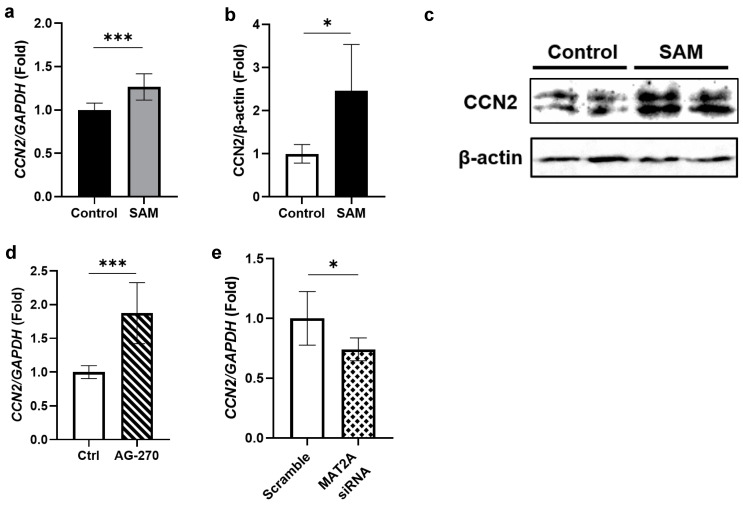
Effect of SAM on the gene expression and protein level of CCN2 in HCS-2/8 cells. HCS-2/8 cells were placed in 6-well plates at a density of 6 ×10^5^ cells/well and allowed to settle for 24 h prior to the subsequent experiments. (**a**) Exogenous SAM enhanced the gene expression of CCN2 (n = 9). Cells were treated with SAM (10 μg/mL) for 3 days, then total RNAs were extracted and subjected to RT-qPCR. (**b**,**c**) The addition of SAM enhanced the protein level of CCN2. Cells were cultured with SAM (10 μg/mL) for 6 days, then cell lysates were prepared and analyzed by western blotting (**c**) with an antibody specific to CCN2. (**b**) Quantitative values of western blots were calculated from the density of the CCN2 signals and normalized to the signals of β-actin (n = 5). (**d**)The effect of AG-270 on the gene expression of *CCN2* in HCS-2/8 cells. Cells were treated with AG-270 (1 μM) or DSMO, for 3 days and RT-qPCR showed that AG-270 significantly increased the gene expression of CCN2 (n = 9). (**e**) The effect of siRNA against *MAT2A* on the gene expression of *CCN2* in HCS-2/8 cells. Cells were transfected with siRNA against MAT2A (80 pmol) or scramble siRNA by lipofectamine for 24 h and subjected to RNA extraction and RT-qPCR. The result shows that MAT2A knockdown significantly suppressed the gene expression of CCN2 (n = 5). In these graphs, the Y axis presents the fold-change relative to the control group set up at ratio = 1 (Welch’s *t* test, * *p* < 0.05, *** *p* < 0.005).

Subsequently, we examined the gene expression and protein levels of CCN2 under SAM stimulation because CCN2 is known to stimulate the gene expression and production of cartilage-specific ECM in growth plate and articular chondrocytes. As shown in the results, SAM increased both the gene expression (Figure 5a) and protein level of CCN2 (Figure 5b,c). To confirm these findings, we investigated whether inhibiting the synthesis of intracellular SAM with AG-270 would result in a reduction in CCN2 gene expression, or not. However, unexpectedly, AG-270 pre-treatment significantly increased the CCN2 gene expression (Figure 5d). Therefore, we also investigated the effect of siRNA against MAT2A on the CCN2 gene expression and found that siRNA significantly suppressed the gene expression of CCN2 (Figure 5e).

### 2.6. Exogenous SAM Increases Polyamine Levels in Chondrocytes

**Figure 6:**
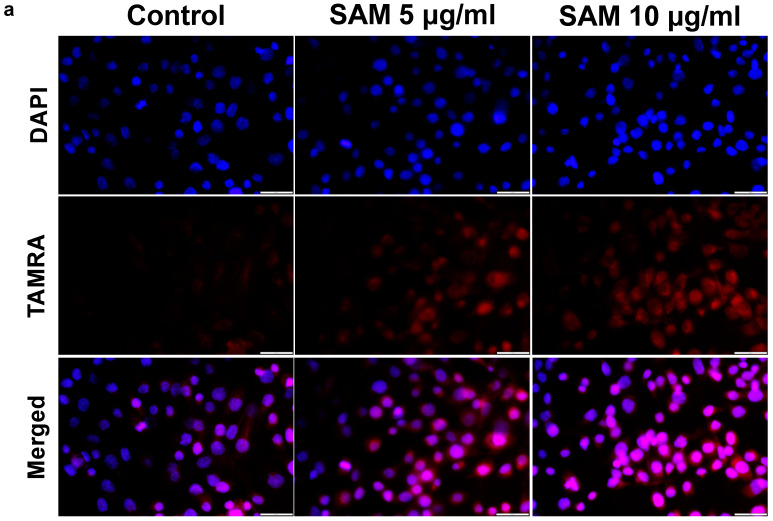

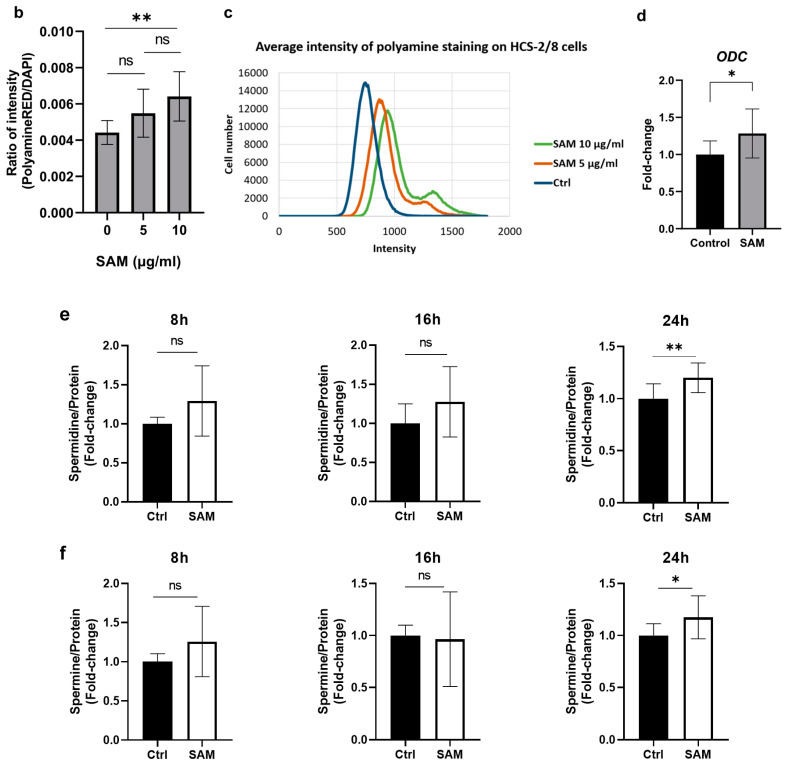
Additional SAM promoted polyamine production in chondrocytes. (**a**) PolyamineRED staining indicated polyamine levels were enhanced by additional SAM in HCS-2/8 cells. HCS-2/8 cells were seeded in 96-well plates at a density of 3000 cells/well and cultured in the presence of SAM (10 μg/mL) for 3 days. Then the cells were stained with PolyamineRED as described in Materials and Methods. The scale bar indicates 500 μm. (**b**) The intensity levels of stained cells shown in (a) were measured with a fluorescent plate reader. (**c**) The histogram of PolyamineRED intensity from each cell (X-axis) versus cell numbers (Y-axis). (**d**) SAM enhanced the expression of ornithine decarboxylase (*ODC*). HCS-2/8 cells (6 × 10^5^ cells/well) were seeded in 6-well plates and incubated with SAM (10 μg/mL) for 3 days. Total RNAs were extracted from cells and subjected to RT-qPCR. The quantitation was normalized to *GAPDH* and the level set in control = 1 (Welch’s *t*-test, * *p* < 0.05, n = 9). (**e**,**f**) An HPLC analysis of spermidine and spermine in HCS-2/8 cells. Cells (6 × 10^5^ cells/well) were seeded in 6-well plates and allowed to adhere for 24 h. Then, SAM was added at the concentrations of 10 μg/mL and incubated for indicated time. Cell lysates were collected at the time indicated and subjected to HPLC as described in Materials and Methods. The levels of spermidine (**e**) and spermine (**f**) were normalized to total protein levels. (Welch’s *t* test, * *p* < 0.05,** *p* < 0.01, n = 6). ns = not significant.

SAM is a precursor of decarboxylated SAM, which in turn donates aminopropyl groups in synthesizing polyamines. To clarify whether exogenous SAM enhances polyamine production in chondrocytes or not, we first detected polyamine levels in HCS-2/8 cells exposed to SAM for 3 days using PolyamineRED^TM^ staining. As a result, the fluorescence intensity of polyamines stained in red in SAM-treated cells was higher than that in the control group (Figure 6a). As shown in Figure 6b,c, a fluorescence plate reader and image analyzer detected that the intensity level of the stained polyamines in the SAM-treated group was higher than that in the control group. Furthermore, expression of *ODC,* the rate-limiting enzyme of polyamine synthesis, was 28.2% higher in the SAM-treated group than in the untreated group (Figure 6d). This trend was consistent between both the HCS-2/8 and RCS cells (Supplementary Figure S5a–c). To validate the findings, HPLC analysis was conducted to quantify the levels of spermidine and spermine in HCS-2/8 cells upon SAM addition (Figure 6e,f). As depicted in Figure 6e,f, the levels of both spermidine and spermine were observed to likely increase 24 h after the SAM addition at a concentration of 10 μg/mL. In addition, an HPLC analysis of RCS cells treated with SAM showed that both spermidine and spermine levels tended to be enhanced when SAM was present at high concentrations (Supplementary Figure S5d,e).

### 2.7. DFMO, an Inhibitor of ODC, Decreases SAM-Induced Aggrecan Production in Chondrocytes

**Figure 7:**
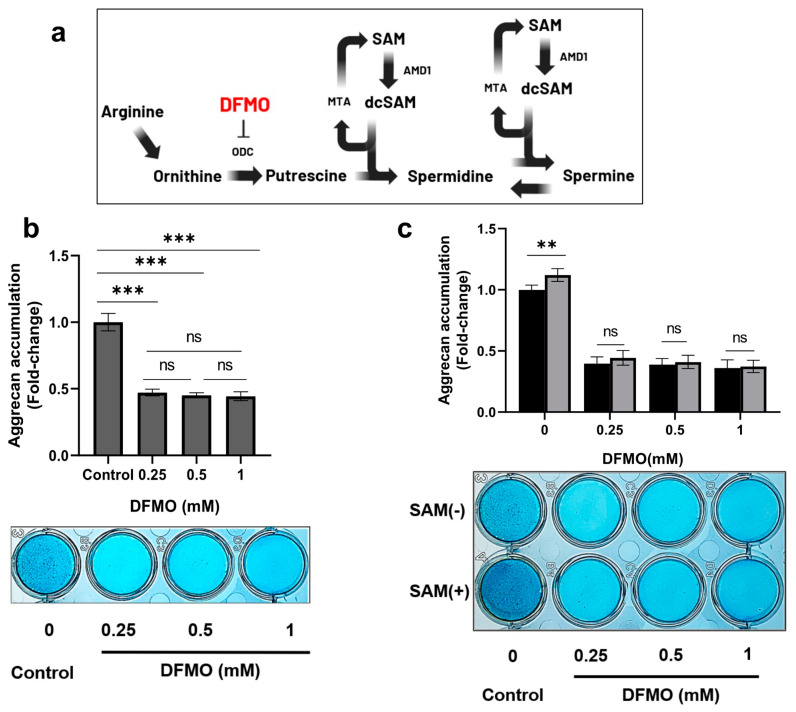
DFMO inhibites the SAM-induced aggrecan accumulation in chondrocytes. (**a**) The position inhibited by difluoromethylornithine (DFMO) in polyamine biosynthesis pathway. (**b**) Aggrecan accumulation was drastically reduced by DFMO. HCS-2/8 cells (1 × 10^5^ cells/well) were seeded in 24-well plates and allowed to adhere for 24 h. Then cells were cultured with DFMO at the indicated concentrations for 14 days. Alcian blue staining was performed to measure the aggrecan accumulation. (*** *p* < 0.005, One-way ANOVA, Dunnett, n = 6). (**c**) The aggrecan enhancement that occurred with the addition of SAM was suppressed by DFMO. HCS-2/8 cells (10 × 10^4^ cells/well) were seeded in 24-well plates and allowed to adhere for 24 h. Then cells were cultured with/without SAM (10 μg/mL) in the presence of DFMO for 14 days. Alcian blue staining was performed to measure the aggrecan accumulation. (** *p* < 0.01, two-way ANOVA, Tukey, n = 6). ns = not significant. These values represent the relative ratio to the DFMO(-) SAM(-) group as the control (ratio = 1.0).

To further investigate the contribution of polyamine synthesis to the mechanism of SAM action in chondrocytes, we used an inhibitor of the rate-limiting enzyme of polyamine synthesis, which is difluoromethylornithine (DFMO), an ornithine decarboxylase inhibitor (Figure 7a) [32]. As shown in Figure 7b, DFMO drastically reduced the production of aggrecan by more than 50% in HCS-2/8 cells in comparison to the control group. Additionally, the enhanced aggrecan accumulation induced by SAM was completely eliminated in the presence of DFMO (Figure 7c). These findings indicated that SAM-induced aggrecan production is dependent on polyamine synthesis.

### 2.8. Inhibition of Polyamine Synthesis by DFMO Eliminates SAM-Induced Enhancement of Gene Expression in HCS-2/8 Cells

**Figure 8:**
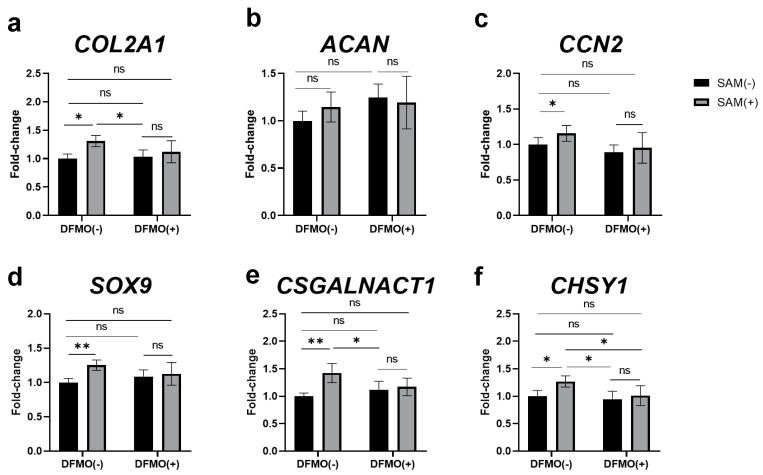
DFMO inhibited the SAM-induced enhancement of the gene expression in HCS-2/8 cells. HCS-2/8 cells (6 × 10^5^ cells/well) were seeded in 24-well plates and allowed to adhere for 24 h. Then cells were treated with DFMO (0.5 mM) with/without SAM (10 μg/mL) for the next 3 days. Subsequently, total RNAs were extracted from the cells and the expression of (**a**) *COL2A1*, (**b**) *ACAN,* (**c**) *CCN2*, (**d**) *SOX9,* (**e**) *CSGALNACT1*, and (**f**) *CHSY1* was analyzed by RT-qRCR. In these experiments, SAM(-) DFMO(-) was used as control. In these experiments, SAM(-) DFMO(-) was used as control (* *p* < 0.05, ** *p* < 0.01, two-way ANOVA, Tukey, n = 6). ns = not significant.

The findings from Alcian blue staining shown in Figure 7 suggest a possibility that the regulation of the gene expression induced by SAM in chondrocytes is controlled via polyamine synthesis. Therefore, we next investigated whether DFMO eliminated the SAM-induced enhancement of the expression of chondrogenesis-related and cartilage marker genes, or not. As shown in Figure 8, DFMO decreased the expression of almost all the genes investigated. In addition, as indicated in Supplementary Figure S6, the effect of SAM on the expression of some genes was eliminated. These results indicate that polyamines at least partially mediate these gene regulations, playing an essential role in the mechanism underlying SAM’s action in chondrocytes. 

## 3. Discussion

In this study, we provide insights into the effects of SAM on chondrocyte differentiation. Previously, Harmand et al. reported that treatment with SAM at a concentration of 10 μg/mL resulted in a significant increase in cartilage-specific proteoglycan (now called aggrecan) determined via ^3^H-serine and ^35^S-sulfate incorporation, as well as hexuronic acid in human articular osteoarthritis chondrocytes, but did not reveal its mechanisms [14]. In this study, Alcian blue staining demonstrated that SAM enhanced sulfated aggrecan accumulation, a typical component of cartilaginous proteoglycan [33] in HCS-2/8 cells, with the most significant improvement occurring at a concentration of 10 μg/mL (Figure 1a–c). This finding is consistent with the results of the aforementioned study. We also confirmed our results by conducting the same experiments with RCS cells, and a comparable result was observed: the administration of SAM at 100 μg/mL significantly enhanced the aggrecan production (Supplementary Figure S1a,b). In addition, we found that administering SAM externally resulted in a positive outcome regarding the gene expression of aggrecan core protein, type II collagen, two major markers of cartilaginous ECM, and the chondrogenesis marker SOX9 in HCS-2/8 cells (Figure 2a–c) and RCS cells (Supplementary Figure S2a,b,f). These findings indicated that SAM has the potential to enhance proteoglycan production and chondrocyte differentiation. 

Our current study indicates that SAM stimulated proteoglycan production at high concentrations (50 or 100 μg/mL for RCS and 5 or 10 μg/mL for HCS-2/8 cells). However, the administration of SAM at these concentrations had an inhibitory effect on cell proliferation in both HCS-2/8 (Figure 1d,e) and RCS cells (Supplementary Figure S1c,d) without any appreciable damage during culture in the presence of SAM. These phenomena are consistent with a few reports indicating that reported that SAM at high concentrations had a negative effect on cell proliferation [34,35,36]. Taken together, these reports and our results suggest that cell cycle arrest generally occurs as the first step in the differentiation process [37]. The contrasting effects of SAM in terms of cell proliferation and aggrecan accumulation imply that the enhancing effect of SAM on proteoglycan production is independent from cell proliferation.

Notably, GAG is an important structure for the function of the ECM as a cushion, and chondroitin sulfate (CS) constitutes approximately 80% of GAGs found in adult articular cartilage [38]. These facts prompted us to evaluate whether SAM has an impact on the gene expression of CS synthesis enzymes in chondrocytes, similar to that of aggrecan core protein. Interestingly, quantitative RT-PCR revealed that the addition of exogenous SAM enhanced the expression of glycosyltransferases; *CHSY1*, *CHSY3*, *CSGALNACT1*, and *CSGALNACT2* in HCS-2/8 cells (Figure 2d–g). Considering the positive effect of SAM observed in Alcian blue staining together, these findings indicate that SAM has a positive effect on ECM production via the gene induction of, not only the aggrecan core protein, but also the enzymes responsible for the synthesis of chondroitin sulfate, especially *CSGALNACT1*, which has been suggested to mainly regulate CS synthesis in cartilage [39,40].

Endogenous SAM is described as a natural compound present that occurs in all living cells, participates in diverse biochemical reactions, and is involved in an extensive network over many crucial processes [41]. Therefore, we assumed that endogenous SAM might play a role in the regulation of the cartilage-specific phenotype. In the current study, we observed that the inhibition of intracellular SAM biosynthesis by AG-270 led to a significant drop in aggrecan production and the expression of cartilage-associated genes in HCS-2/8 (Figure 3c and Figure 4a–e) and RCS cells (Supplementary Figures S3b,c and S4a–f). These experiments were designed and carried out to evaluate the impact of exogenous SAM, as well as to confirm the role of endogenous SAM in regulating chondrocyte function. Collectively, these results were not merely conformable with the increase in proteoglycan synthesis observed through Alcian blue staining, but could also elucidate the chondroprotective effect that was mentioned in a previous in vivo study [42].

Our study showed that exogenous SAM enhanced the gene expression and the protein level of CCN2 in HCS-2/8 cells (Figure 5a–c). These findings suggest that CCN2 may participate in the mechanism by which SAM stimulates the chondrocyte differentiation. This was also supported by the findings that MAT2A siRNA and AG-270 suppressed CCN2 gene expression in HCS-2/8 and RCS, respectively (Figure 5e, Supplementary Figure S4c). However, AG-270 did not decrease, but rather unexpectedly increased the gene expression of *CCN2* in HCS-2/8 (Figure 5d). Previous studies have reported that the expression of CCN2 is well correlated with tissue repair [43,44,45], including cartilage regeneration [29,46]. Because of these reports, it is speculated that high concentrations of AG-270 may cause cell damage in HCS-2/8, and that the increase in the *CCN2* induction may have been triggered as a response to counteract the impairment. To clarify the precise role of CCN2 under SAM treatment, more comprehensive explorations are needed.

Along with its main function as a common methyl donor, SAM is also involved in the polyamine synthesis pathway as an aminopropyl-group donor. In the early stages, ornithine is decarboxylated by ODC enzyme to produce putrescine, whereas SAM is decarboxylated to dcSAM by the adenosylmethionine decarboxylase 1 (AMD1) enzyme. Putrescine uses an aminopropyl group from dcSAM to produce spermidine and spermine. Therefore, it is quite convincing that a high concentration of polyamines was detected using PolyamineRED™ staining in SAM-treated HCS-2/8 cells (Figure 6a,b) and RCS cells (Supplementary Figure S5a,b). An HPLC analysis showed that spermine and spermidine levels were upregulated in SAM-treated cells compared to those in non-treated cells, respectively (Figure 6d,e, Supplementary Figure S5d,e). Interestingly, SAM also promoted the gene expression of the rate-limiting enzyme ODC for polyamine synthesis in both HCS-2/8 (Figure 6c) and RCS cells (Supplementary Figure S5c). These data revealed a new insight that SAM plays dual roles in the polyamine synthesis; one is a dcSAM supplier and the other is a promoter of ODC gene expression. 

Next, to investigate the contribution of polyamine synthesis to the mechanism underlying the functions of SAM, we used DFMO to completely inhibit polyamine synthesis by interfering with ODC, the rate-limiting enzyme of polyamine synthesis. The results showed that DFMO suppressed aggrecan production in chondrocytes, as well as reduced SAM-induced aggrecan accumulation (Figure 7c). This result indicates that polyamine synthesis plays an important role in the SAM-induced ECM production. 

As shown in Figure 8, the presence of DFMO did not result in a decrease in the expression of the genes of interest in HCS-2/8. This indicates that the expression of these genes at basal levels do not depend on polyamine synthesis. Interestingly, DFMO abolished the positive effect of additional SAM on the expression of all genes in HCS-2/8, which demonstrates that the stimulation of these gene expressions by additional SAM is dependent on polyamine synthesis.

Overall, the endogenous SAM and exogenous SAM showed partially similar behavior, but some results indicated differences in their effects. This is possibly due to the fact that the concentration of SAM in serum is reported to be in the hundreds of nanograms per milliliter [47], which is considerably lower than the concentration of SAM that was added in this study. Since the concentration of SAM administered as a supplement or drug may be close to the concentration used in the experiments conducted here, the results of this study may help to understand the conditions that may occur in vivo when these drugs are taken.

Although we provided preliminary results on the effects of SAM on the chondrocytic phenotype, more in vivo experiments are necessary to obtain definitive conclusions. In this study, we identified two pathways as candidates that mediate the effects of SAM on chondrocytes; enhancement of polyamine production and stimulation of the gene expression of the cartilage regeneration factor CCN2. However, the responses of RCS and HCS-2/8 cells to the experimental conditions were different from each other. This means that more experimental models must be created to further confirm our hypotheses.

In conclusion, this study elucidated the mechanism of the positive effect of SAM on chondrocytes, that is, stimulation of the gene expression of CCN2, SOX9, and cartilage ECM-related factors and an increase in polyamine synthesis, both of which may interact with each other. 

## 4. Materials and Methods

### 4.1. Materials

Dulbecco’s modified Eagle’s medium (DMEM), and fetal bovine serum (FBS) were purchased from Nissui Pharmaceutical Co. Ltd. (Tokyo, Japan) and Nichirei Bioscience, Inc. (Tokyo, Japan), respectively. S-adenosylmethionine (SAM), alpha-difluoromethylornithine (DFMO), and Protease Inhibitor Cocktail (#P8340) were purchased from Sigma-Aldrich (Tokyo, Japan). AG-270 was purchased from Selleck Chemicals (Houston, TX, USA).

### 4.2. Cell Culture

The human chondrosarcoma-derived chondrocytic cell line (HCS-2/8) [46], and rat chondrosarcoma-derived chondrocytic cell line (RCS) [48] were grown in DMEM supplemented with 10% FBS, 50 μg/mL streptomycin, and 100 units/mL penicillin G and 5% FBS. All cells were maintained in a humidified incubator (5% CO_2_ atmosphere) at 37 °C until they reached confluence. The culture medium was changed every 3 days. 

### 4.3. Proliferation Assay

For the cell counting assay, chondrocytes were seeded in 96-well plates and exposed to SAM [49]. After 2 days, the cell proliferation kit-8 (Dojindo, Tokyo, Japan) was used to measure cell growth according to the manufacturer’s instructions. Cell proliferation was measured by measuring the optical absorbance at a wavelength of 450 nm. For counting the number of cells, the cells were cultured as same as the cell counting assay, and cell nuclear were stained with 1 μg/mL 4,6-diamidino-2-phenylindole (DAPI; Sigma, Tokyo, Japan) for 30 min. The number of nuclear was counted as the number of cells by using the Cellomics Array Scan^TM^ High Content Screening System (Thermo Fischer Scientific, Waltham, MA, USA). 

### 4.4. Alcian Blue Staining

Alcian blue staining was performed to measure the accumulation of aggrecan [50]. Briefly, the cells were fixed with 3% cold acetic acid and immediately stained with 1% Alcian blue 8GX (Sigma, Tokyo, Japan) for 30 min at room temperature. The plates were then washed 3 times with distilled water to remove excess dye. Guanidine chloride (6 M) was added to extract bound Alcian blue dye and incubated overnight at room temperature. Absorbance at a wavelength of 620 nm was measured using a plate reader.

### 4.5. Reverse-Transcription—Quantitative PCR

Total RNA was isolated from the cells using the RNeasy Kit (Qiagen, Hilden, Germany) according to the manufacturer’s instructions. Isolated total RNA was reverse-transcribed to cDNA using a Takara RNA PCR kit (AMV), version 3.0 (Takara Shuzo, Tokyo, Japan). Next, PCR with cDNA was performed in duplicate using a SYBR^®^ Green Real-time PCR Master Mix (Toyobo, Tokyo, Japan) and StepOne^TM^ (Applied Biosystems, Foster City, CA, USA) [51]. For all quantitative PCR measurements, the total amount of cDNA was standardized based on the expression level of Glyceraldeyde-3-phostphate dehydrogenase (GAPDH). The nucleotide sequences of the primers used in this study are listed in Table 1 and Supplementary Table S1.

### 4.6. Western Blotting

Cells in each well were harvested by using a scraper and radio immunoprecipitation assay (RIPA) buffer containing Protease Inhibitor Cocktail. After sonication for 10 s and centrifugation at 12,000× *g* for 10 min, protein solutions were collected as supernatants. The protein concentration in each sample was detected using a Pierce BCA Protein Assay Kit (Thermo Fischer Scientific). The protein solution adjusted to the same concentration was applied to 10% SDS-PAGE, and then transferred to a polyvinylidene difluoride membrane (Bio-Rad Laboratories, Hercules, CA, USA). The experimental protocol has been previously described [52]. The proteins of CCN2 and MAT2A were detected with anti-CCN2 antibody (ab6992) (Abcam PLC, Cambridge, UK), anti-MAT2A (NB110-94158) (Novus Biologicals LLC, Centennial, CO, USA). To detect the β-actin protein, mouse anti-beta Actin, Monoclonal Antibody (010-27841) (FUJIFILM Wako Pure Chemical Corporation, Osaka, Japan) and Alexa Fluor^TM^ 680 goat anti-mouse IgG were used. The images were acquired and calculated by ChemiDoc^TM^ Imaging system (Bio-Rad). The expression levels of each of the proteins are represented as a value relative to that of β-actin.

### 4.7. Knockdown of mRNA Using MAT2A siRNA

Cells were seeded in 6-well plates at the indicated concentrations and allowed to adhere onto the plate for 24 h. Then, the cells were transfected with 80 pmol of siRNA targeting MAT2A (sc-149292) (Santa Cruz) using Lipofectamine RNAiMAX (Thermo Fischer Scientific) following the manufacturer’s instructions. Scrambled siRNA (sc-37007) was used as control. After 24 h, the cells were then harvested and subjected to subsequent experiments [53]. The knockdown efficiency of MAT2A siRNA was evaluated by western blotting and RT-qPCR.

### 4.8. Detection of Polyamine Level by PolyamineRED Staining

Chondrocytes were seeded at the indicated concentrations in 96-well plates and exposed to SAM for 3 days. At the time of staining, the cells were treated with 30 μM Polyamine RED dye for 1 h. After incubation, the cells were washed 3 times with phosphate-buffered saline (PBS), followed by DAPI staining and formalin fixation. Fluorescence images and quantitative analysis were performed with a fluorescent plate reader and the Cellomics Array Scan^TM^ High Content Screening System in according to the manufacturer’s instructions.

### 4.9. Determination of SAM Level by ELISA

The cells were harvested by a scraper and sonicated on ice in 500 μL of PBS. After centrifugation at 12,000× *g* for 10 min at 4° C, the supernatant was removed for ELISA. The SAM ELISA was performed using a SAM ELISA kit (Cell Biolabs, Inc., San Diego, CA, USA) in accordance with the manufacturer’s instructions.

### 4.10. Determination of Polyamine Level by High Performance Liquid Chromatography

Cells were seeded at a density of 60 × 10^4^ cells/well in 6-well plates and treated with SAM for the indicated time. Then, the cells were harvested by using a scraper with cold PBS. Cells were homogenized with sonication and centrifuged at 10,000× *g* at 4 °C. Supernatants were collected and stored at −30 °C until use. Polyamines were quantified by dansylation and HPLC, as reported previously [54,55]. Briefly, the supernatant was incubated with 150 nM 1,8-diaminooctane as an internal standard and 4.3% PCA for 30 min on ice. After centrifugation at 12,000× *g* for 15 min at 4 °C, the resulting supernatant was incubated with 600 µL of 10 mg/mL dansyl chloride and 600 µL of saturated Na_2_CO_3_ for 30 min at 60 °C, then incubated with 100 µL of 100 mg/mL l-proline for 30 min at 60 °C. Dansyl derivatives were extracted with 1.5 mL of toluene and evaporated in a centrifugal vacuum concentrator, Speedvac Plus (Savant Instruments, Inc., Holbrook, NY, USA). The resulting pellet was resuspended with 100 µL of solvent (acetonitrile:water = 7:3). A HPLC technique using the Chromaster system (Hitachi High-Tech Co., Tokyo, Japan) was used to detect the polyamine content.

### 4.11. Statistical Analysis

All experiments were repeated at least twice, and similar results were obtained. All data are expressed as the mean ± standard deviation (SD). Mean values were compared using Welch’s *t*-test, one-way ANOVA, or two-way ANOVA, and post hoc comparisons were performed if necessary.

## Figures and Tables

**Table 1:** Nucleotide sequences of primers for HCS-2/8.

Gene	Sequences	Species
CCN2	F-GCAGGCTAGAGAAGCAGAGC R-ATGTCTTCATGCTGGTGCAG	Human
ACAN	F-TTCGGGCAGAAGAAGGAC R-CGTGAGCTCCGCTTCTGT	Human
COL2a1	F-ATGGCTTCCAGAGGCC GAC R-TTGCTGCATGAGTTGCCACGCA	Human
ODC	F-ATGGCTTCCAGAGGCC GAC R-TTGCTGCATGAGTTGCCACGCA	Human
SOX9	F-AGGCAAGCAAAGGAGATGAA R-TGGTGTTCTGAGAGGCACAG	Human
GAPDH	F-GCCAAAAGGGTCATCATCTC R-GTCTTCTGGGTGGCAGTGAT	Human
CHSY1	F-CGACAGGAACTTTCTCTTCGTGG R-GGTACAGATGTGTCAGAACCCTC	Human
CHSY3	F-AGTTGGAGCGGGCTTACAGTGA R-CAGCACCTCAAAGCGAGAGTGT	Human
CSGALNACT1	F-GGATGACGTGTCAGTATCGGTC R-CCGTACCACTATGAGGTTGCTG	Human
CSGALNACT2	F-TCATCTCACAGTGGTGTATTTTGG R-GCACCCACATTTAGTCCTCGTC	Human

## Data Availability

Data are contained within the article.

## Supplementary Materials

The following supporting information can be downloaded at: https://www.mdpi.com/article/10.3390/ijms242417294/s1.

## Abbreviations

ACAN, Aggrecan; AMD1, Adenosylmethionine Decarboxylase 1; CCN2, Cellular communication network factor 2; CTGF, Connective tissue growth factor; *COL2A1,* Collagen type 2a1; DFMO, Difluoromethylornithine; CHSY, Chondroitin sulfate synthase; CSGALNACT, Chondroitin sulfate N-acetylgalactosaminyltransferase; dcSAM, decarboxylated SAM; DMEM, Dulbecco’s modified Eagle’s medium; ECM, Extracellular matrix; ELISA, Enzyme-linked immunosorbent assay; FBS, Fetal bovine serum; GAG, Glycosaminoglycan; GAPDH, Glyceraldeyde-3-phostphate dehydrogenase; HCS-2/8, Human chondrocyte-like cell line-2/8; HPLC, High Performance Liquid Chromatography; MAT2A, Methionine adenosyltransferase 2A; ODC, Ornithine decarboxylase; PBS, phosphate-buffered saline; RCS, Rat chondrosarcoma derived cell line; SAM, S-adenosylmethionine; siRNA, Small interfering RNA; SOX9, Sry-Box transcription factor 9
