# Supplementary material for: Positive Regulation of S-Adenosylmethionine on Chondrocytic Differentiation via Stimulation of Polyamine Production and the Gene Expression of Chondrogenic Differentiation Factors
Source: Int J Mol Sci. 2023 Dec 9;24(24):17294. doi: 10.3390/ijms242417294 (PMC10743985; doi:10.3390/ijms242417294)
Supplement: Supplementary file 1 [file ijms-24-17294-s001.zip › Supplementary (Figure legends and Table).pdf]

Figure S1. SAM enhances aggrecan production in RCS cells without promoting proliferation. RCS cells ($6\times10^4$ cells/well) were seeded into 24-well plates and allowed to adhere for 24 h. After adding SAM to the culture at the indicated concentrations, the cells were incubated for 7 days. (a) Alcian blue staining showed an increase in aggrecan accumulation in the SAM-treated groups after 7 days of culture. (b) Quantitative measurement of Alcian blue staining in RCS culture with 7-day SAM stimulation showed that at concentrations of 50 and 100 μg/ml SAM significantly enhanced aggrecan accumulation (values are indicated as fold-change relative to the non-treated group, one-way ANOVA, Dunnett, ***p<0.005, n=12). (c) Cell counting assay in cultures treated with several concentrations of SAM. RCS cells (1,500 cells/well) were inoculated into 96-well plates and allowed to adhere for 24 h. Different concentrations of SAM were then added to the wells. After 2 days, cell proliferation was measured using a WST-8 assay. The Y-axis indicates the relative ratio of optical absorbance obtained at a wavelength of 450 nm to the control (ratio=1) (one-way ANOVA, Dunnett, *p<0.05, n=3). (d) Counting the number of cells cultured with SAM at several time points. The cell number was counted at each day within three days of RCS culture with 100 μg/ml SAM. Cell numbers between the SAM-treated group and the control group at each time point were comparatively analyzed. (Welch's t-test, ***p<0.005, n=10).

Figure S2. SAM enhances the gene expression of cartilage-specific markers, chondrogenesis associated factors and enzymes involved in chondroitin sulfate synthesis in RCS cells. RCS cells were seeded in 6-well plates at a density of $3\times10^5$ cells/well and incubated with SAM at a concentration of 10 μg/ml. After 3 days, total RNAs were extracted from the cells and subjected to RT-qPCR to evaluate the effects of SAM stimulation on the gene expression of cartilage markers: *Acan* (a), *Col2a1* (b), chondrogenesis associated factor: *Sox9* (c), *Ccn2* (d), enzymes involved in chondroitin sulfate synthesis: *Chsy1* (e), and *Csgalnact1* (f). (Values represent fold-change relative to the untreated control group, Welch's t-test, *$p<0.05$, **$p<0.01$, n=9).

Figure S3. Inhibiting intracellular SAM synthesis suppresses aggrecan accumulation in RCS cells. (a) AG-270 pretreatment decreased intracellular SAM levels in RCS cells. Cells ($6\times10^4$ cells/well in 6-well plates) were treated with AG-270 at the indicated concentrations for 3 days, and cell lysates were subjected to an ELISA (one-way ANOVA, Dunnett, n=3). (b) Alcian blue staining revealed that AG-270 pretreatment suppressed aggrecan accumulation in RCS cells. (c) Quantification of Alcian blue staining showed that AG-270 significantly reduced aggrecan production in RCS cells (one-way ANOVA,

Dunnett, n=3). In these experiments, the values represent the fold-change relative to the non-treated groups (ratio=1).

Figure S4. Effect of AG-270 on the induction of the gene expression by SAM in RCS cells. RCS cells were inoculated at $3\times10^5$ cells/well in 6-well plates and allowed to adhere for 24 h. Then, AG-270 (2 μM) was introduced to RCS cells. After 3 days, total RNAs were extracted from the cells, and RT-qPCRs were performed. (a) Col2a1, (b) Acan. (c) Ccn2, (d) Chsy1, (e) Csgalnact1, (f) Sox9. The expression level of each gene was normalized to that of Gapdh. These values were relative to the AG-270(-) group as the control (ratio=1.0). (Two-way ANOVA, Tukey, *$p<0.05$, ***$p<0.005$, n=9).

Figure S5. Exogenous SAM promotes polyamine production in the chondrocytic cell line. RCS cells were seeded in 96-well plates at a density of 1,500 cells/well and cultured in the presence of SAM (100 μg/ml) for three days. (a) PolyamineRED staining revealed polyamine levels in the RCS cells. Scale bar, 500 μm. (b) The histogram of polyamineRED intensity from each cell (X-axis) versus cell number (Y-axis) (n=3). (c) RT-qPCR showed that SAM pretreatment enhanced the gene expression of Odc. RCS cells ($3\times10^5$ cells/well) were seeded in 6-well plates and incubated with SAM (100

μg/ml) for 3 days. Total RNAs were extracted from the cells and subjected to RT-qPCR. The data were normalized to those of *Gapdh*, and the expression level of the control was set to 1 (Welch's t-test, *$p<0.05$), n=9. (d-e) An HPLC analysis revealed that SAM treatment tended to enhance the levels of spermidine and spermine in RCS cells. RCS cells ($3\times10^5$ cells/well) were inoculated in 6-well plates and allowed to adhere for 24 h. SAM was then added at the indicated concentrations, and cells were collected after 3 days. Cell lysates were subjected to high-performance liquid chromatography (HPLC) as described in the Materials and Methods. Spermidine and spermine levels were normalized to the protein levels. The results showed that spermidine and spermine levels tended to be increased by SAM in comparison to the non-treated groups. (one-way ANOVA, Dunnett's test, n=3).

Figure S6. Effect of DFMO on the SAM-induced gene expression in RCS cells. RCS cells ($3\times10^5$ cells/well) were seeded into 24-well plates and allowed to adhere for 24 h. The cells were then pre-treated with DFMO (5 mM) with or without SAM (100 μg/ml) for the next 3 days. Total RNAs were extracted from the cells and expression of (a) Col2a1, (b) Acan, (c) Odc, (d) Sox9, (e) Csgalnact1, (f) Chsy1 was analyzed by RT-qPCR. The gene expression of cartilage markers (*Col2a1* and *Acan*) was suppressed by DFMO, regardless

of the presence of SAM. SAM(-) DFMO(-) was used as the control (*p<0.05, two-way ANOVA, Tukey, n=3).

Table S1. Nucleotide sequences of primers for RCS.

| Gene | Sequences | species |
|---|---|---|
| Ccn2 | F-GCAGGCTAGAGAAGCAGAGC<br>R-ATGTCTTGATGCTGGTGCAG | Rat |
| Acan | F-TTGGAGCCGGAGACGACAGA<br>R-AGAGGCAGAGGGACTTTCGGT | Rat |
| Col2a1 | F-TTCCTCCGTCTACTGTCCACTGA<br>R-CTACATCATTGGAGCCCTGGAT | Rat |
| Odc | F-CCT GAG ACC TTC GTG C<br>R-GCT GAT GCG ACG TAG T | Rat |
| Sox9 | F-AGACCAGTACCCGCATCT<br>R-CGCTCCGCCTCCTCCAC | Rat |
| Gapdh | F-GCCAAAAGGGTCATCATCTC<br>R-GTCTTCTGGGTGGCAGTGAT | Rat |
| Chsy1 | F-CGACAGGAACTTTCTCTTCGTG<br>R-AGTGTGTCTGGTCTTATGAGATGC | Rat |
| Csgalnact1 | F-GCCTCGCAGGAGGGAAAGTT<br>R-GAGGAAAACGAGACCCCAAGCA | Rat |
