# Supplementary figures and images for: Positive Regulation of S-Adenosylmethionine on Chondrocytic Differentiation via Stimulation of Polyamine Production and the Gene Expression of Chondrogenic Differentiation Factors
Source: Int J Mol Sci. 2023 Dec 9;24(24):17294. doi: 10.3390/ijms242417294 (PMC10743985; doi:10.3390/ijms242417294)

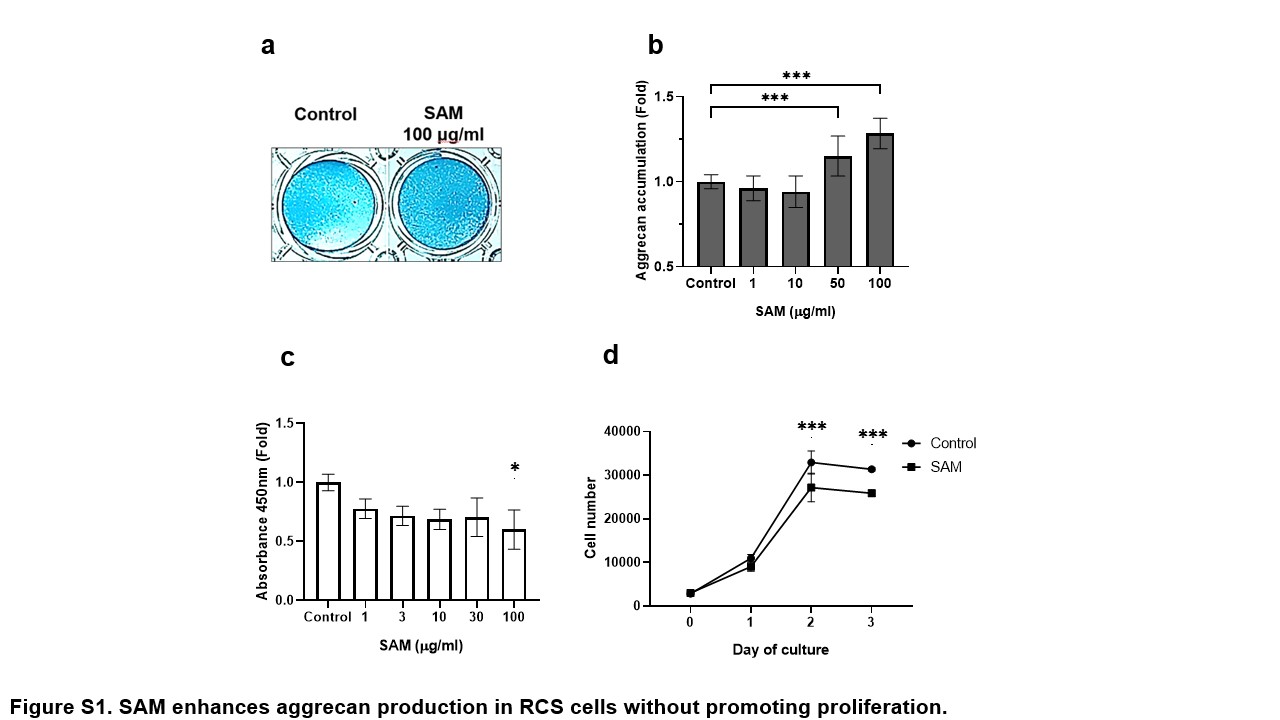

Supplement: Supplementary file 1 [file ijms-24-17294-s001.zip › Slide1.jpg]

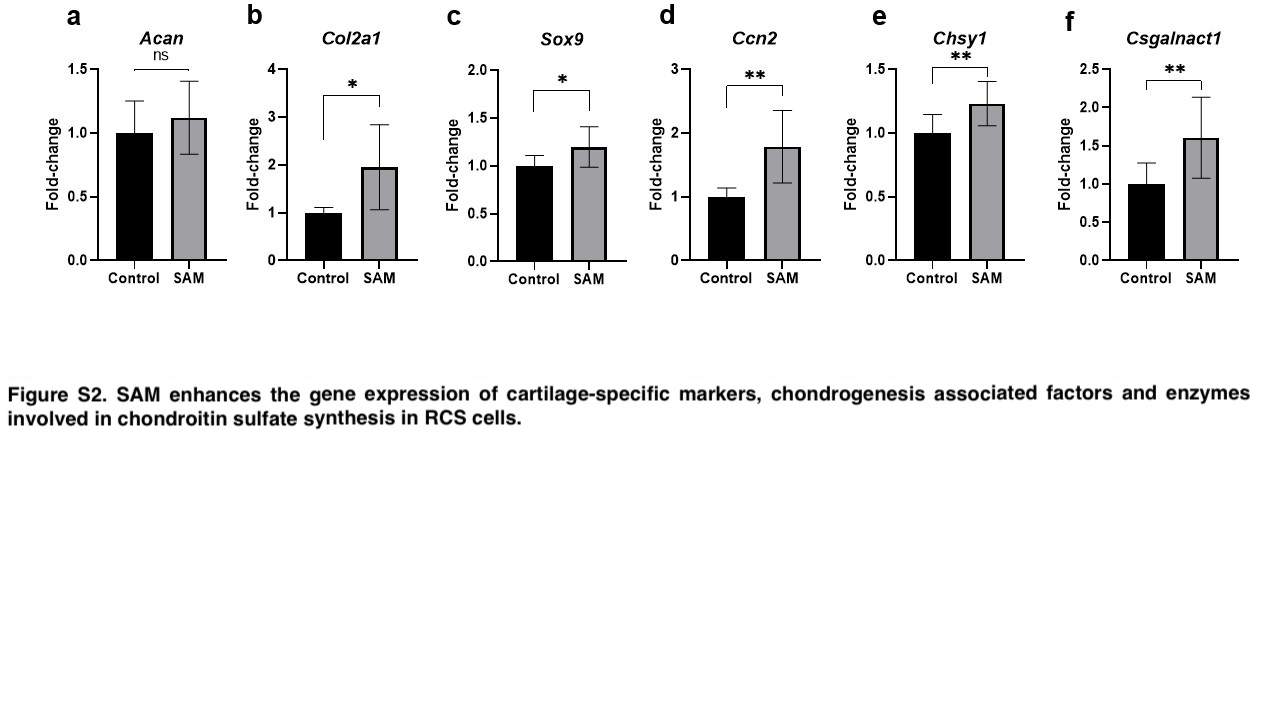

Supplement: Supplementary file 1 [file ijms-24-17294-s001.zip › Slide2.jpg]

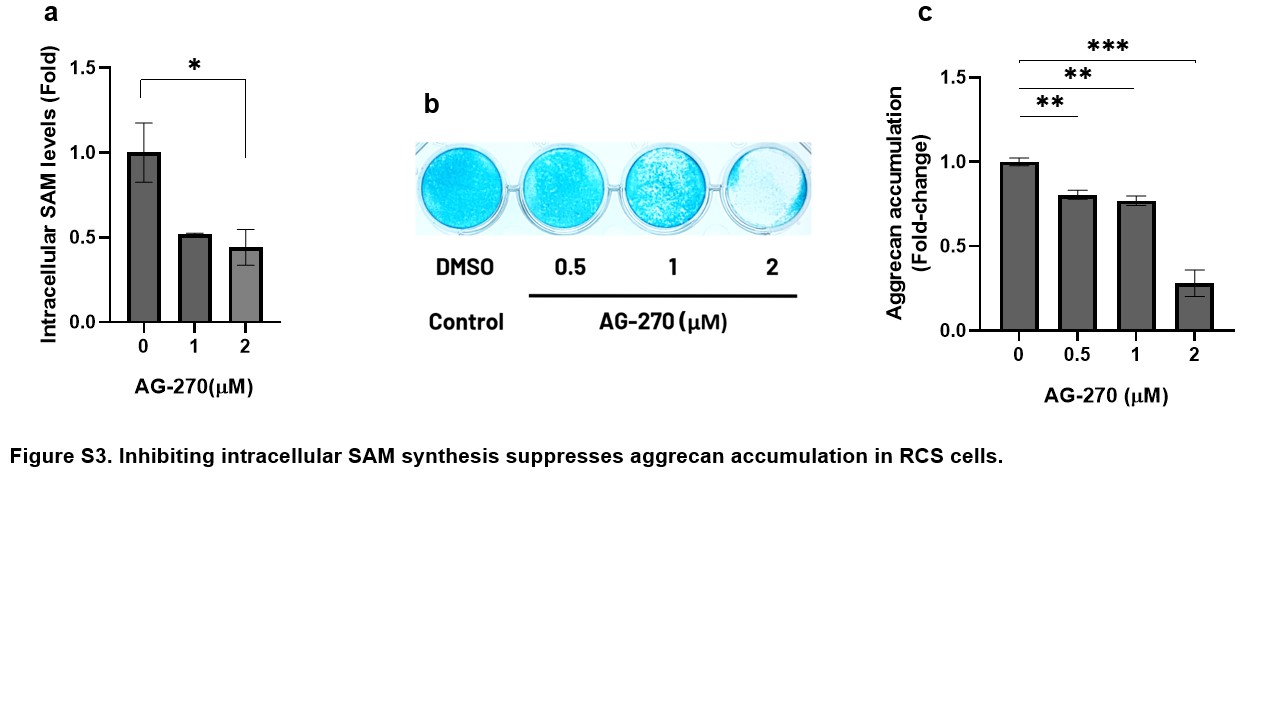

Supplement: Supplementary file 1 [file ijms-24-17294-s001.zip › Slide3.jpg]

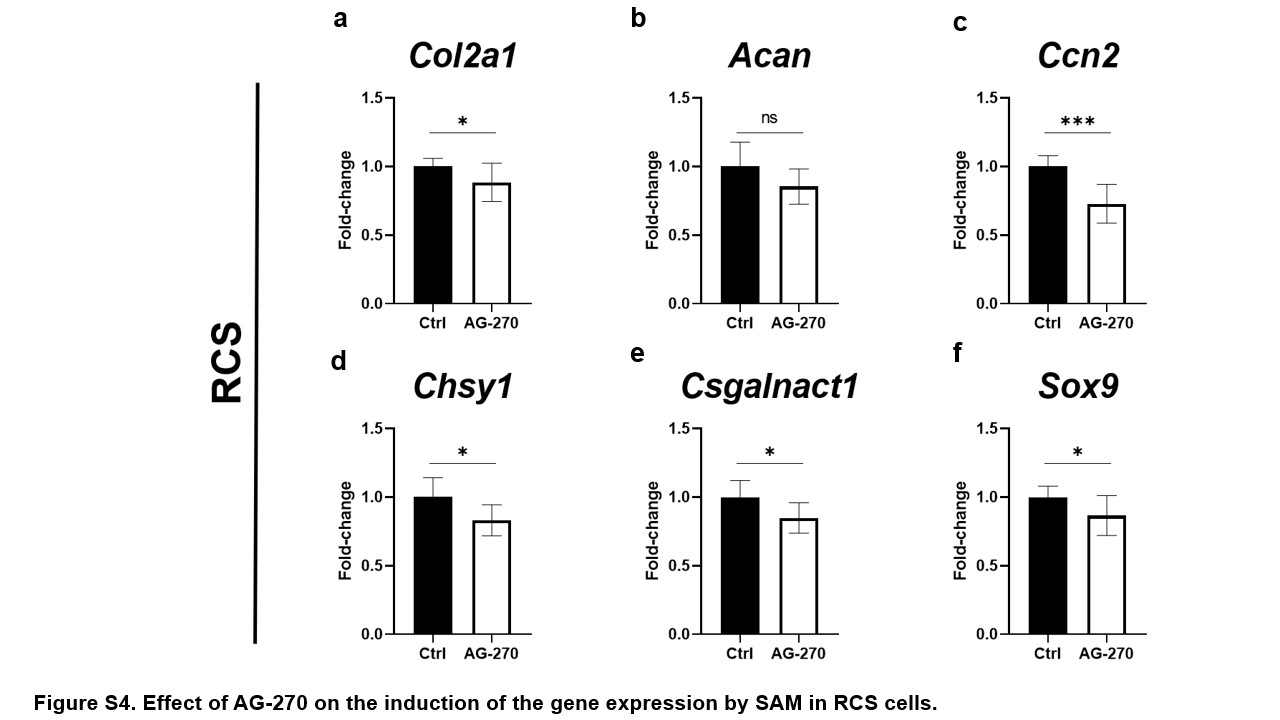

Supplement: Supplementary file 1 [file ijms-24-17294-s001.zip › Slide4.jpg]

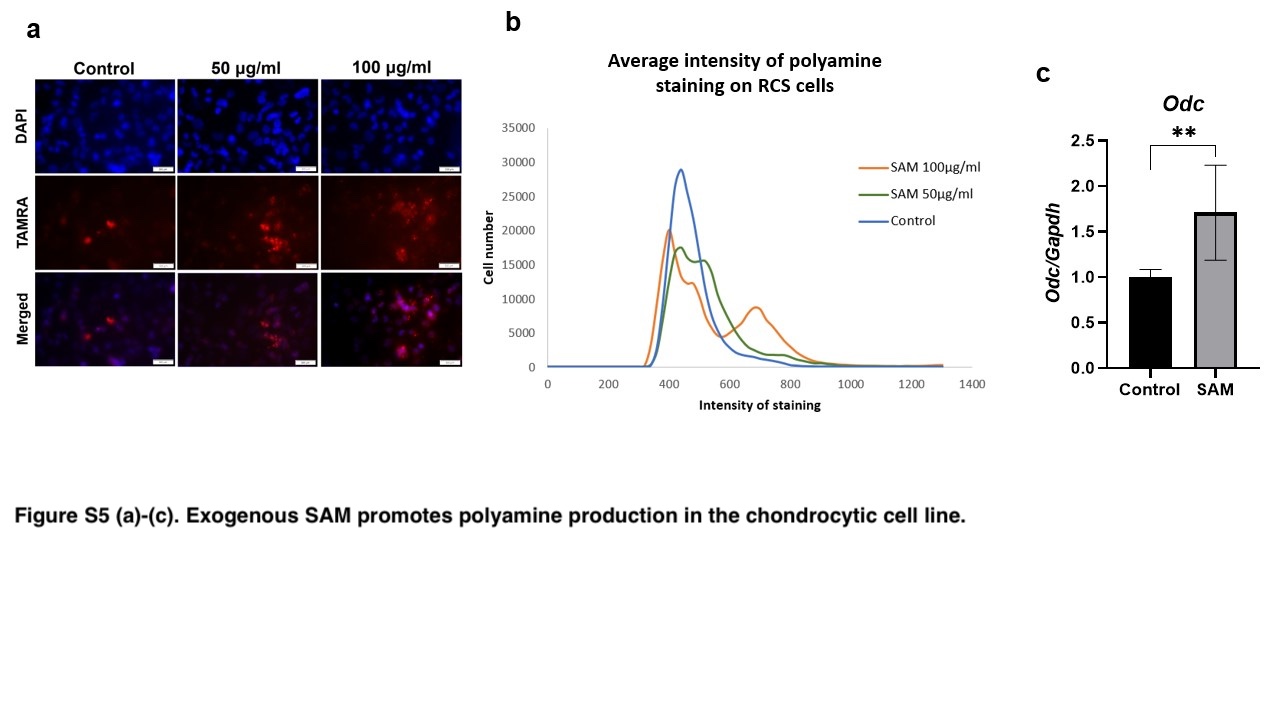

Supplement: Supplementary file 1 [file ijms-24-17294-s001.zip › Slide5.jpg]

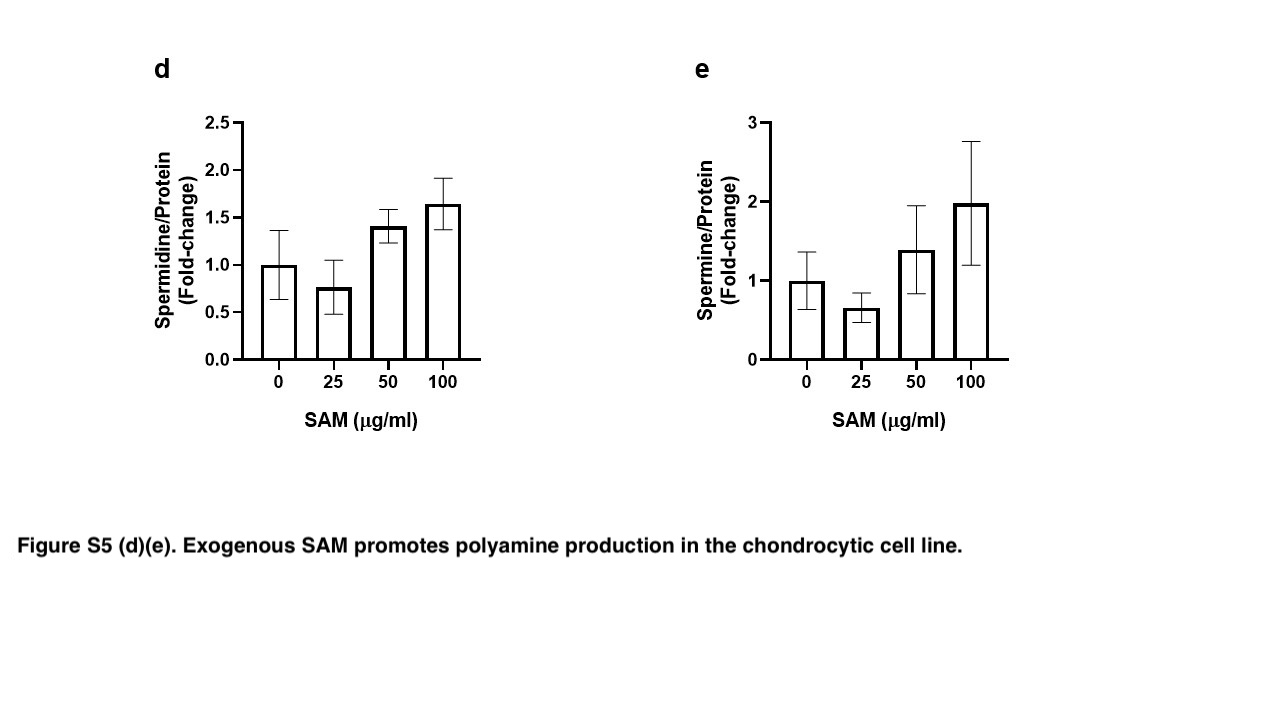

Supplement: Supplementary file 1 [file ijms-24-17294-s001.zip › Slide6.jpg]

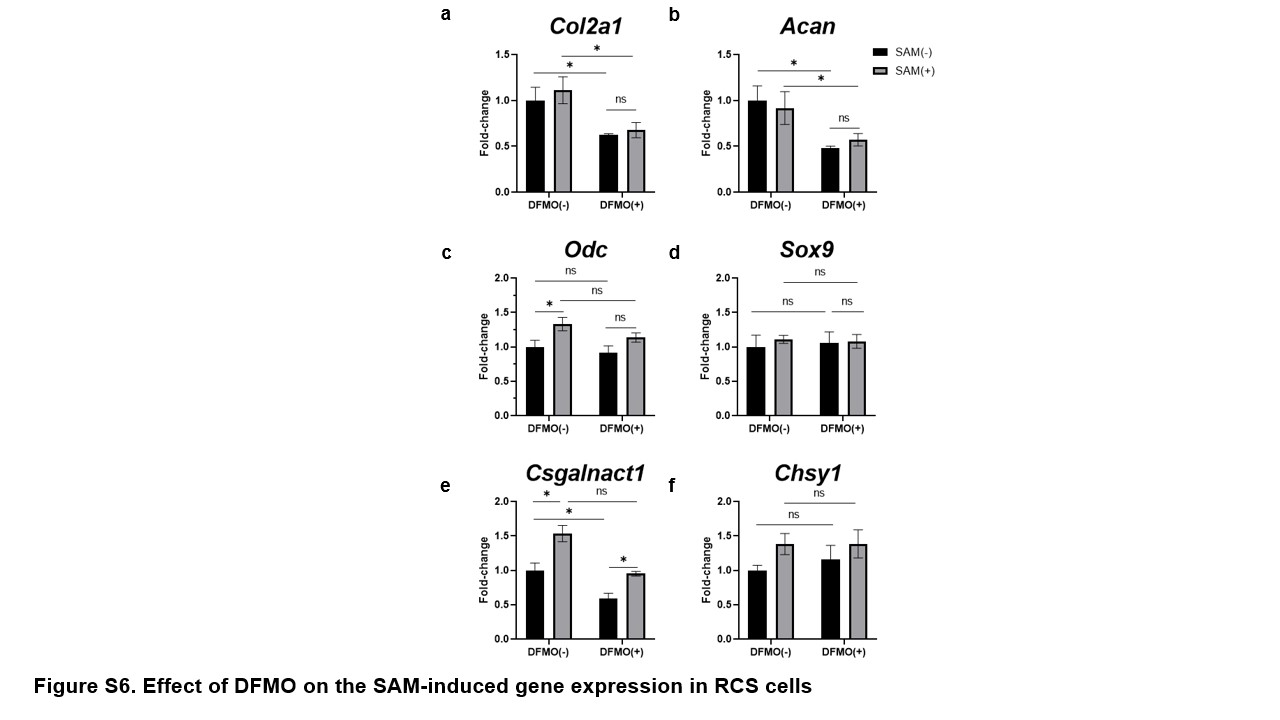

Supplement: Supplementary file 1 [file ijms-24-17294-s001.zip › Slide7.jpg]
